# Immunotherapy revolutionizing brain metastatic cancer treatment: personalized strategies for transformative outcomes

**DOI:** 10.3389/fimmu.2024.1418580

**Published:** 2024-07-29

**Authors:** Ting Li, Shichen Sun, Yubing Li, Yanyu Zhang, Linlin Wei

**Affiliations:** ^1^ Medical Oncology Department of Thoracic Cancer 1, Cancer Hospital of China Medical University, Liaoning Cancer Hospital & Institute, Shenyang, Liaoning, China; ^2^ Department of Radiotherapy, Cancer Hospital of China Medical University, Liaoning Cancer Hospital & Institute, Shenyang, Liaoning, China

**Keywords:** metastatic brain tumors, immunotherapy, immune checkpoint inhibitors, BBB, cancer

## Abstract

Brain metastatic cancer poses a significant clinical challenge, with limited treatment options and poor prognosis for patients. In recent years, immunotherapy has emerged as a promising strategy for addressing brain metastases, offering distinct advantages over conventional treatments. This review explores the evolving landscape of tumor immunotherapy in the context of brain metastatic cancer, focusing on the intricate interplay between the tumor microenvironment (TME) and immunotherapeutic approaches. By elucidating the complex interactions within the TME, including the role of immune cells, cytokines, and extracellular matrix components, this review highlights the potential of immunotherapy to reshape the treatment paradigm for brain metastases. Leveraging immune checkpoint inhibitors, cellular immunotherapies, and personalized treatment strategies, immunotherapy holds promise in overcoming the challenges posed by the blood-brain barrier and immunosuppressive microenvironment of brain metastases. Through a comprehensive analysis of current research findings and future directions, this review underscores the transformative impact of immunotherapy on the management of brain metastatic cancer, offering new insights and opportunities for personalized and precise therapeutic interventions.

## 1 Introduction

Recent advancements in diagnostic and therapeutic modalities have significantly improved survival rates in patients with malignant tumors. However, the development of symptomatic brain metastases (BM) presents a considerable challenge and remains a leading cause of mortality in these patients. Brain metastatic carcinoma, characterized by the metastasis of malignant tumors from other parts of the body to the skull, represents one of the most prevalent intracranial tumors encountered in clinical practice (1, 2). This condition infiltrates various intracranial tissues, including the brain parenchyma, spinal cord membranes, nerves, and capillaries, with brain parenchymal metastasis being the most frequent. Consequently, patients often experience epilepsy, cognitive dysfunction, sensory impairment, motor dysfunction, and cranial nerve damage, leading to a significant decline in their overall quality of life (3). The primary treatment options for metastatic brain tumors currently encompass surgical resection and radiation therapy. While conventional chemotherapeutic agents have shown limited efficacy in the central nervous system (CNS), the emergence of targeted small molecule tyrosine kinase inhibitors has revolutionized therapeutic approaches by effectively crossing the BBB and displaying activity within the CNS (4). Additionally, immunotherapy has emerged as a promising treatment modality for brain metastatic carcinoma, overcoming the traditional challenges associated with pharmacotherapy by demonstrating intracranial activity. Notably, two major factors impeding pharmacotherapy efficacy in BM include the unpredictable molecular profiles of BM relative to the primary tumor and their variable responsiveness to drugs, alongside the constraints posed by limited drug penetration through the human BBB and blood-tumor barrier (BTB) (5).

The tumor immune microenvironment (TME) plays a crucial role in tumor development, progression, and metastasis. It consists of tumor-associated immune cells and associated cytokines (6, 7). Tumor cells are a prime component of the immune microenvironment and can hinder immune responses by releasing various molecules and cytokines, allowing them to escape immune surveillance (8). Meanwhile, diverse immune cells play essential roles in mounting an effective immune response. Additionally, fibroblasts, vascular endothelial cells, and other cell types contribute to the construction of the TME and influence tumor cell growth and metastasis. Interleukins (ILs), tumor necrosis factors (TNFs), and transforming growth factors (TGFs) are pivotal in regulating immune responses and tumor growth (9). Immunotherapy, the manipulation of the body’s immune system to enhance its ability to target and eliminate tumor cells, intimately links with the tumor immune microenvironment (10). On one hand, the tumor immune microenvironment greatly influences the effectiveness and response rates of immunotherapy. For instance, certain tumor cells employ the upregulation of programmed death-ligand 1 (PD-L1) to evade attacks from immunotherapy by inhibiting T cell activation and cytotoxicity. On the other hand, immunotherapy actively influences tumor growth and metastasis by modulating the immune microenvironment. For instance, targeting the cytotoxic T-lymphocyte-associated protein 4 (CTLA-4) molecule promotes T cell activation and cytotoxicity, thereby inhibiting tumor growth and metastasis.

In recent years, rapid advancements in biomedical science have led to novel ideas for immunotherapy in the treatment of malignant brain metastases. This article provides a comprehensive summary of research pertaining to various immunotherapies for brain metastatic cancer. Additionally, it outlines recent advances in immunotherapy for brain metastatic cancer in conjunction with the specific tumor immune microenvironment found in brain metastatic foci.

## 2 Brain-specific structures and microenvironment

### 2.1 BBB

**Figure 1:**
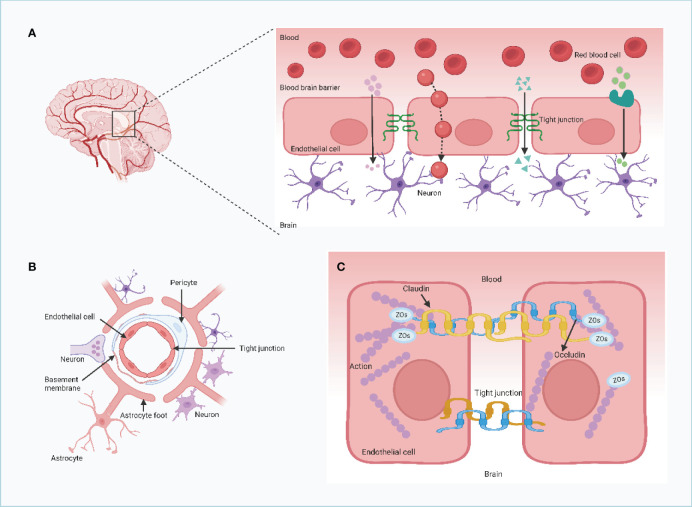
The structure and feature of the blood brain barrier (BBB). **(A)** Claudins and occludin compress two neighboring endothelial cells together. These proteins are connected to cytoskeletal proteins through auxiliary proteins such as ZO, which promote the formation of TJs. The blood-brain barrier is formed by endothelial cells connected by tight junction proteins (TJs) and separates the brain from components of the circulating blood. **(B**, **C)** The capillary lumen of the blood-brain barrier is surrounded by endothelial cells, and TJs are located between the endothelial cells of the brain thus preventing the flow of most substances from the blood into the brain. TJs allow essential nutrients to enter the brain parenchyma by simple diffusion, passive diffusion between cells, and transport proteins that transport essential macromolecules, but they limit the entry of potentially harmful molecules from the blood into the brain.Endothelial and pericytes are surrounded by a common basement membrane. The ends of astrocytes surround the endothelium and pericytes and provide the connection between neurons and the blood-brain barrier. (Created with BioRender.com).

The BBB consists of a heterogeneous composition of cell types, including microvascular endothelial cells, the basement membrane, and adjacent astrocytes Among these cell types, microvascular endothelial cells serve as the primary constituents of the BBB, which are interconnected by tight junction proteins such as TJP-1 (ZO-1), claudin, and occludin, forming a cohesive barrier (**Figure 1**) (11). These tight junction proteins are crucial for maintaining cellular interconnectivity and preventing the entry of macromolecules into the neural tissue Moreover, the basement membrane plays a vital role in the establishment and maintenance of the BBB by establishing adhesive connections between microvascular endothelial cells and astrocytes via proteins, polyamines, and sugars (12, 13). The adjacent astrocytes, being a specialized subtype of glial cells, not only provide structural support but also influence neuronal activity (14). Functionally, the BBB selectively filters substances from the bloodstream, permitting the passage of only small, specific molecules. This “selective permeability” feature of the BBB is crucial for the stable functioning of neural tissue (15). However, after the onset of brain metastases, the integrity of the BBB is disrupted to various extents, leading to the formation of what is referred to as the BTB. Experimental models of brain metastases have revealed that the BTB tends to be more permeable to drugs and contrast media than the BBB.

The BBB, on the other hand, is the barrier between plasma and brain cells formed by the walls of brain capillaries and glial cells, and between plasma and cerebrospinal fluid formed by the choroid plexus (16, 17). During tumor treatment, circulating tumor cells may enter the brain and form brain metastases. However, because of the presence of the BBB, most circulating tumor cells cannot pass through, limiting the occurrence of BM to some extent (18). Recent studies have shown that circulating tumor cells disrupt the integrity of the BBB, thereby promoting the development of brain metastases (19). Circulating tumor cells may disrupt the BBB by interacting with endothelial and pericyte cells of the BBB and releasing substances such as VEGF that alter the permeability of the BBB (20). Circulating tumor cells can also directly invade the endothelial and pericyte cells of the BBB, thereby inducing BBB disruption (21). In addition, circulating tumor cells can further promote the development of BM by interacting with glial cells and inducing their activation, thereby releasing substances that alter the permeability of the BBB, such as interleukin-8 (IL-8) (22, 23). There is growing evidence that MMPs play a role in disrupting tight junctions and promoting tumor brain metastasis. During cerebral ischemia, MMP2 and MMP9 are activated and degrade tight junction proteins such as claudin-5, occludin, and ZO-1 in brain microvascular endothelial cells, increasing the permeability of the blood-cerebrospinal fluid barrier and causing brain edema and hemorrhage (24).

Treatment options for encephalopathy often involve the use of lipid-soluble drugs with low polarity. Studies have demonstrated that the rate at which different drugs enter the brain and cerebrospinal fluid from the blood can vary greatly. Drugs that have a high binding affinity to plasma encounter difficulties in traversing the BBB and reaching brain tissue. Under normal physiological conditions, drugs that exist in a non-dissociated form are more likely to pass through the BBB, while drugs with a high oil-water partition coefficient tend to have easier access to the CNS. In normal circumstances, the presence of the BBB provides a protective effect for the CNS, limiting the entry of certain substances from the bloodstream into the brain. The restrictive properties of the BBB are more pronounced in comparison to the capillaries of other organs. The BBB acts as a barrier, allowing essential substances for brain metabolism to pass through while preventing the entry of foreign matter, such as bacteria and viruses, thereby safeguarding the brain tissue from potential harm. The invasion of viruses and bacteria into the central nervous system typically occurs through dissemination via the bloodstream, necessitating passage through the BBB. Thus, the integrity of the BBB contributes to determining the occurrence and severity of CNS infections. Moreover, the existence of the BBB reinforces the stability of brain cells and enhances the resistance of brain tissue to environmental changes, thereby promoting the organism’s adaptive capacity. In pathological conditions, the functioning of the BBB becomes compromised. For instance, in cases of brain tumors, substances such as 32P or other fluorescent materials that normally have limited BBB permeability can penetrate the brain tissue. This principle is applied in radiological diagnoses of brain tumors. Notably, more than 89% of experimental brain metastatic lesions exhibit some disruption in BBB permeability, varied in terms of significance, with only 10% achieving therapeutic drug concentrations. Therefore, increasing BBB permeability to peripheral immune cells and antitumor drugs has become a crucial area of focus within therapeutic research for brain metastatic cancer.

### 2.2 Immune microenvironment of metastatic brain tumors

The TME is a complex and intricately coordinated system consisting of multiple components. These include tumor cells, immune cells, inflammatory cells, tumor-associated fibroblasts (TAMs), neighboring mesenchymal tissue, microvasculature, as well as various cytokines and chemokines (25). The TME can be further divided into two distinct compartments: the immune microenvironment, predominantly governed by immune cells, and the non-immune microenvironment, primarily controlled by fibroblasts. Within the TME, there exist intricate interactions and regulatory relationships among its diverse constituents, which exert considerable influence on numerous processes such as tumorigenesis, metastasis, and drug resistance. Furthermore, these complex components contribute to the metabolic heterogeneity of the TME and dictate the behavior of immune cells. Tumor metastasis represents a significant hurdle in tumor management and is a leading cause of mortality in many cancer patients. One key driver of tumor metastasis is the establishment of pre-metastatic niches (PMNs), which are specific locations where a microenvironment conducive to tumor metastasis forms. Remarkably, certain tumor cells exhibit organ-specific tropism, suggesting the selective nature of tumor metastasis. The formation of PMNs is mediated by a tripartite interplay involving tumor-derived secreted factors (TDSFs), extracellular vesicles (EVs), and bone marrow-derived cells (BMDCs). Specifically, TDSFs and EVs derived from primary tumors induce the recruitment of BMDCs to target organs, thereby facilitating the creation of an inflammatory microenvironment conducive to tumor metastasis. Consequently, this inflammatory milieu promotes tumor cell colonization, survival, and growth within PMNs. 

**Figure 2:**
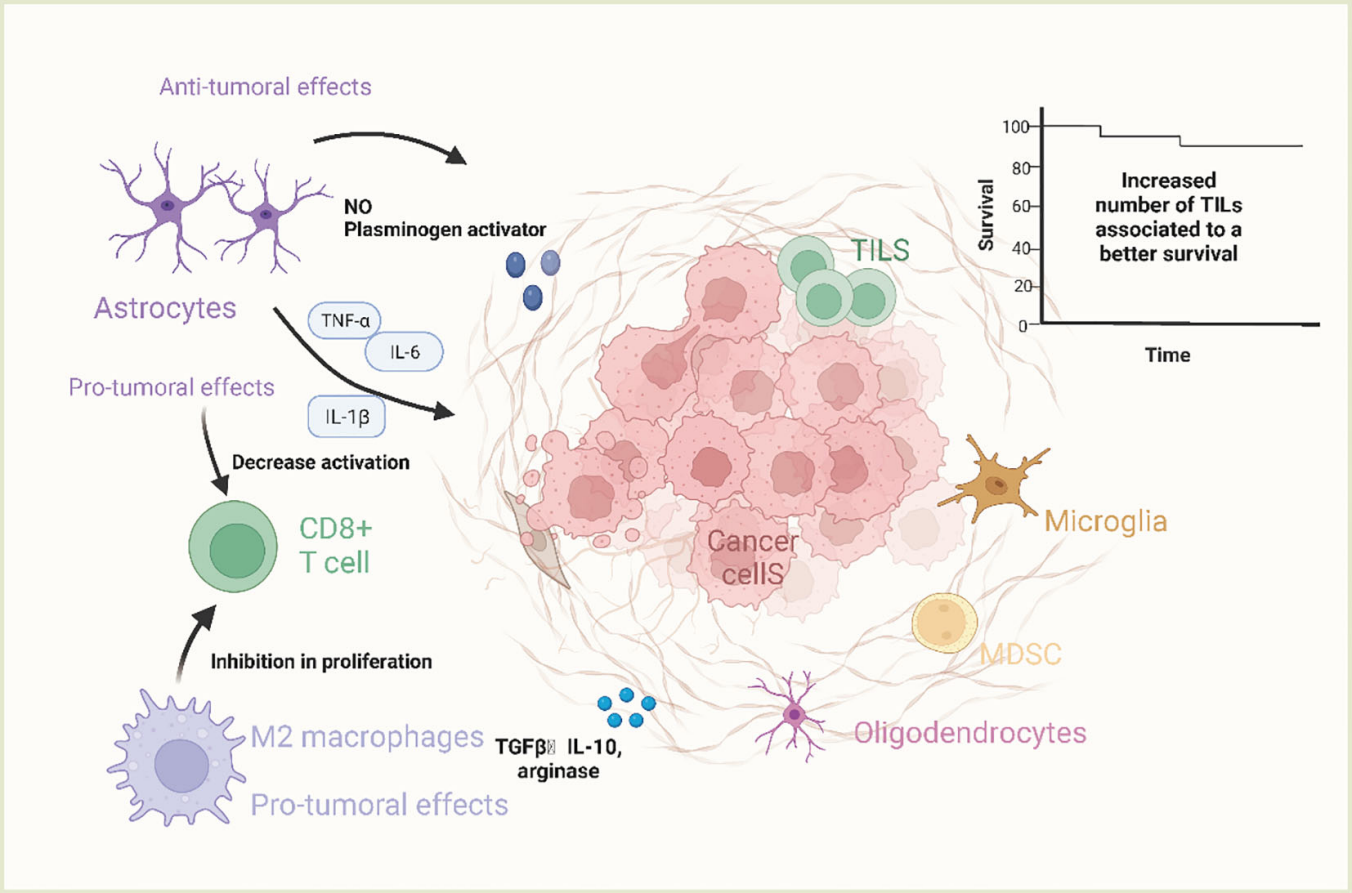
The characteristics of tumor microenvironment of brain metastases. Microglia are resident macrophages in the tumor microenvironment of brain metastases and are not of bone marrow origin. Only when the blood-brain barrier is disrupted can bone marrow-derived macrophages reach the CNS and act as a response to CNS disturbances. These cells are known as tumor-associated macrophages (TAMs), in which M1-like macrophages are usually pro-inflammatory and are stimulated by Toll-like receptor ligands as well as IFN-g and TNF-a. They exert tumor suppressor functions by producing factors such as IL-1, IL-12 and nitric oxide. M2-like macrophages are anti-inflammatory and can be activated by IL-4 and IL-13 to produce molecules such as =TGF-b, arginase, IL-10, and pro-fibrotic factors. M2-like macrophages can also be associated with tumor-promoter functions by inhibiting the proliferation of CD8+ T-cells.M2-like macrophages can also be associated with tumor-promoter functions by inhibiting CD8+ T-cell proliferation.M1-like macrophages are usually pro-inflammatory and stimulated by Toll-like receptor ligands as well as IFN-g and TNF-a. (Created with BioRender.com).

Brain metastasis shares similarities with metastasis in other organs, as it follows a highly selective, nonrandom, multistep process. Tumor cells act as the “seeds” of metastasis, with the microenvironment of the metastatic site serving as the “soil” for their growth (26). The genetic and biological heterogeneity of tumor cells from different subclones within a primary tumor influences their metastatic potential, with only subclones possessing strong invasive properties capable of crossing the blood-cerebrospinal fluid (BSF) barrier to establish brain metastases (27, 28). The brain provides a unique microenvironment featuring astrocytes, stromal cells, cytokines, vascular networks, and metabolic components that either promote or inhibit tumor growth, influencing the development and progression of brain metastasis. Upon hematogenous dissemination, tumor cells interact with brain endothelial cells, secreting cytokines like vascular endothelial growth factor (VEGF) and matrix metalloproteinases (MMPs) that modify the brain microenvironment to support tumor growth (29, 30). Additionally, some researchers suggest that tumor cells may carry activated tumor-associated fibroblasts, acting as part of the microenvironment for brain metastasis initiation, survival, and proliferation. The dynamic interplay between tumor cells and the brain microenvironment ultimately leads to the rapid and irreversible growth of brain metastases. For a detailed illustration of the TME characteristics in brain metastasis, refer to **Figure 2**.

The immune microenvironment of brain metastatic lesions is notably distinct from that of metastatic or primary lesions in other anatomical sites. The brain harbors distinctive microenvironmental characteristics, such as the BBB, specialized environmental cells (including microglia, astrocytes, oligodendrocytes, and neurons), a lymphoid system draining to the neck, and an extracellular matrix. Furthermore, the brain exhibits a unique immunological profile (31, 32). Despite the limited presence of immune cells in the normal brain microenvironment, there is documentation of CNS immune surveillance through CD4^+^ T lymphocytes and CD8^+^ T lymphocytes in healthy individuals. Notably, astrocytes assume a crucial role in the microenvironment of brain metastatic lesions (33). In the initial phases of brain metastatic lesion development, astrocytes predominantly participate in inhibiting the survival of metastatic tumor cells. However, upon the establishment of metastatic tumor cells in the brain, they release a multitude of cytokines that facilitate the polarization of astrocytes from type M1 (tumor suppressor) to type M2 (tumor activator), thereby leading to a significant involvement of astrocytes in promoting tumor cell proliferation (34).

### 2.3 Drivers of brain metastasis development and treatment challenges

The development of brain metastases is influenced by various factors, including tumor cell invasive and metastatic potential, as well as oncogenes and genetic factors (35). The invasive and metastatic potential of tumor cells plays a crucial role in this process. Malignant tumor cells, with their high proliferative and invasive capabilities, are able to breach the cerebral vascular wall and cerebrospinal fluid barrier. Subsequently, they enter the cerebrospinal fluid circulation and establish metastatic foci within the brain. Moreover, tumor cells secrete specific proteins that facilitate their adhesion to brain tissue, enabling their passage through the cerebral vascular wall and successful infiltration into brain tissue via the bloodstream. In addition to tumor cell behavior, the impact of oncogenes and genetic factors on the development of brain metastasis should not be overlooked. Overexpression of cancer genes leads to the emergence of tumors. If primary tumors in other parts of the body are not effectively controlled, cancer genes can be disseminated to the brain via systemic circulation, resulting in intracranial metastases. Certain patients, such as those with neurofibromatosis, retinoblastoma, and angioretinoblastoma, possess a higher susceptibility to familial intracranial metastases. Treatment of brain metastases is complicated by several challenges, including the BBB, drug resistance of tumor cells, and the phenotypic heterogeneity of tumor cells. The BBB serves as a major impediment, preventing many drugs from entering the brain and hampering the efficacy of treatment. Furthermore, tumor cells can acquire resistance to drugs following repeated therapies, thus diminishing the therapeutic impact. The individualized treatment of brain metastases is necessitated by the distinct gene expression profiles and biological properties of various tumor cells. Consequently, an in-depth understanding of tumor cell invasion and metastasis mechanisms, along with a comprehensive comprehension of the physiological and pathological aspects of the BBB, is imperative for the development of more effective treatment strategies and therapeutic agents that can successfully address brain metastasis.

## 3 Immunotherapy for metastatic brain tumors

### 3.1 Immune checkpoint inhibitors

The immune system is known to fulfill a crucial role in safeguarding against cancer (36, 37). This function is commonly referred to as the immune surveillance of tumors, attributing to the immune system’s capacity to recognize and eliminate tumor cells through the recognition of tumor-specific antigens and molecular specificity induced by cell activation (38, 39). The concept of “cancer immune editing” elucidates the dual role of the immune system during tumor pathogenesis, encompassing both host-protective and tumor-sculpting functions (40, 41). The emergence of immune checkpoint inhibitors marks a significant advancement in the realm of tumor therapy, as these inhibitors obstruct autologous tumor antigens, bolster zvex-induced T cell responses, and enhance antitumor effects (42). ICIs are pharmaceutical agents designed to stimulate or augment the immune system’s assault on tumor cells by intervening with specific immune checkpoint molecules. The utilization of immune checkpoint inhibitors necessitates careful consideration of various aspects including the patient’s overall health and ability to manage potential adverse reactions associated with immunotherapy. It is vital to note that not all tumor types are amenable to treatment with immune checkpoint inhibitors, highlighting the importance of selecting the appropriate tumor type for this therapeutic approach. Additionally, understanding the patient’s genetic mutation status is essential, as certain genetic variations may impact the efficacy of immunotherapy. Combining ICIs with other treatment modalities like chemotherapy and radiation therapy can enhance therapeutic outcomes. It is imperative to remain vigilant for potential adverse reactions such as immune-related adverse events, skin issues, and gastrointestinal disturbances, necessitating close monitoring and prompt intervention. Clinical trials with ICIs are shown in **Table 1**.

**Table 1:** Current cancer brain metastasis clinical trials with immune checkpoint inhibitors therapy.

Clinicaltrials.gov	Status	Disease/condition	Intervention/treatment	Main Outcome measures
Identifier			(Drugs)	
NCT04348747	Recruiting	Metastatic TNBC or HER2 + BC	Dendritic cell vaccines with pembrolizumab	Assess mPFS, mOS
				Evaluate the safety of treatment
NCT05629546	Active, not yet recruiting	Advanced or metastatic melanoma	Memory-like natural killer cells with	Progression-free survival
			nivolumab and relatlimab	overall survival
NCT04356222	Recruiting	Leptomeningeal metastases from NSCLC	Durvalumab	Neurological Progression Free Survival (NPFS)
				overall survival
NCT03719768	Active, not yet recruiting	Leptomeningeal metastases	Avelumab	overall survival at 3 months
NCT03025256	Active, not yet recruiting	Leptomeningeal metastases	Nivolumab	overall survival

#### 3.1.1 Adrebrelimab

**Figure 3:**
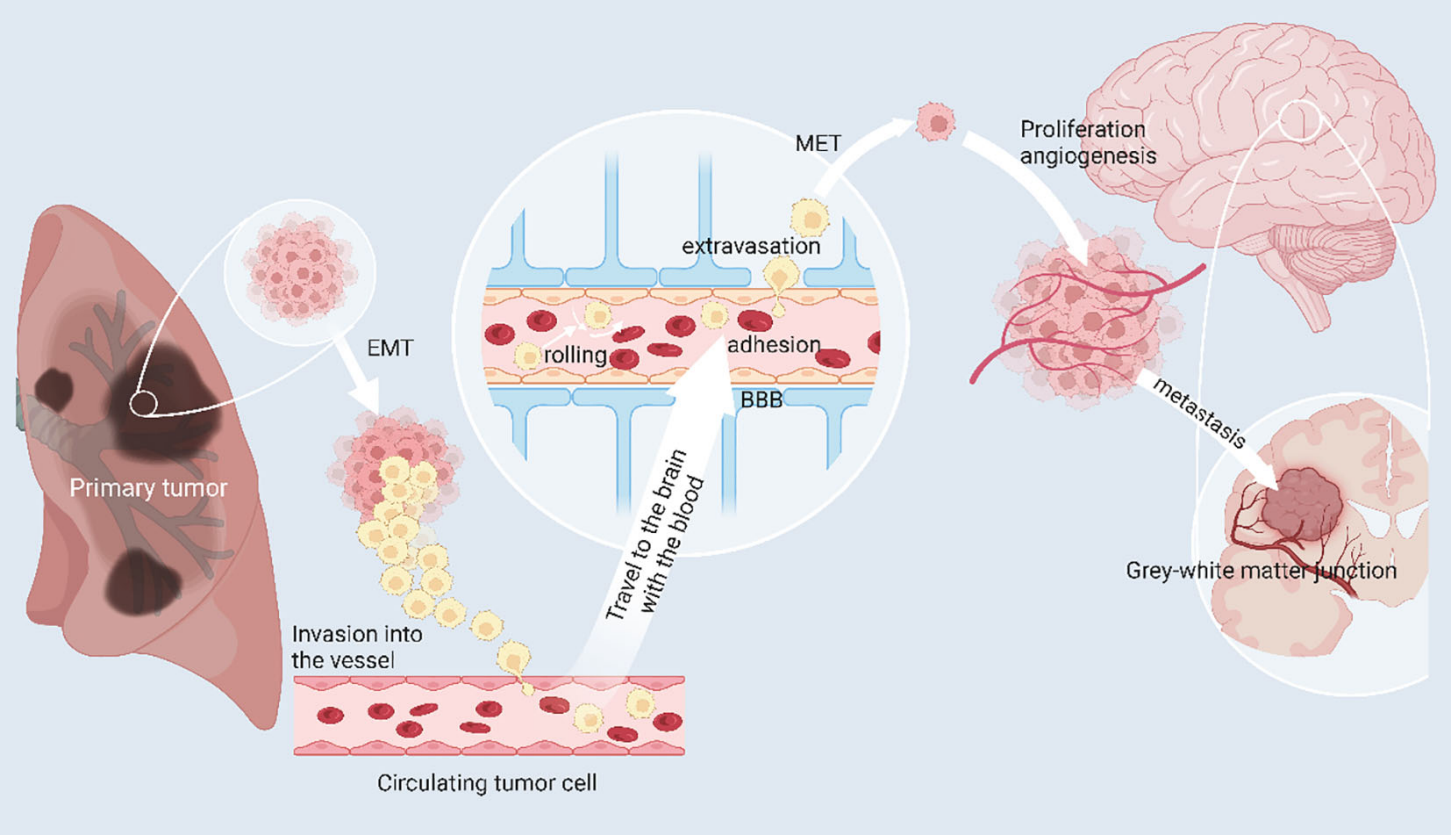
The mechanism of brain metastasis of lung cancer. Primary lung cancer cells can escape from the primary tumor site to invade and circulate in blood vessels, called circulating tumor cells (CTC). In response to chemokines, CTCs can reach the brain and cross the blood-brain barrier by rolling, adherence and extravasation in response to E-ligands and integrins, undergo mesenchymal epithelial transformation (MET) thereby restoring primary tumor properties and generating and adapting to the new tumor microenvironment. Angiogenesis is necessary for the growth of brain metastases. When pure oxygen diffusion is insufficient for the tumor, the tumor can gradually develop a hypoxic microenvironment and promote angiogenesis by overexpressing angiogenesis-stimulating factors. (Created with BioRender.com).

Adrebrelimab is a high-affinity humanized monoclonal antibody against PD-L1 that has demonstrated efficacy and safety in advanced esophageal squamous cell carcinoma (ESCC) (43), extensive SCLC (SCLC) (44), and resectable non-small cell lung cancer (NSCLC) (45). Lung cancer is currently one of the most common malignancies in the world, and its morbidity and mortality are increasing year by year. Local recurrence and metastasis are the main causes of poor prognosis for many lung cancer patients, among which the incidence of brain metastasis reaches 30%~50% (46, 47). NSCLC 20%~50% of patients develop brain metastasis during the course (48, 49) and survive more than 2 years. SCLC The incidence of brain metastasis in patients with SCLC is as high as 60% to 80% (50). The main treatment modalities for BM are whole brain radiotherapy (WBRT), stereotactic radiosurgery (SRS), surgery, and chemotherapy (51). The mechanism of brain metastasis of lung cancer is shown in **Figure 3**. Wang et al (44), evaluated the efficacy and safety of adrebrelimab (SHR-1316) versus standard chemotherapy in the primary treatment of extensive SCLC (ES-SCLC). The main inclusion criteria were age 18-75 years, ES-SCLC patients without a previously confirmed histologic or cytologic diagnosis, and Eastern Cooperative Oncology Group (ECOG) grade 0-1. Ultimately, 230 patients received adrebrelimab combination chemotherapy (adrebrelimab arm) and 232 patients received placebo combination chemotherapy (placebo arm). Patients received carboplatin and etoposide for 4-6 cycles, with concurrent adrebrelimab or corresponding placebo. Adrebrelimab or placebo was administered as maintenance therapy. Results of the research demonstrated a significant improvement in median overall survival in the adrebrelimab group in comparison with the placebo group and an acceptable safety profile, suggesting that this combination treatment may be a new first-line therapy for ES-SCLC.

#### 3.1.2 Atezolizumab

Atezolizumab is a humanized IgG1 anti-PD-L1 drug approved for the treatment of breast cancer (52), SCLC (53), and NSCLC (54). Lin et al (55), reported a case of poorly differentiated adenocarcinoma of the right lobe of the lung treated with atezolizumab monotherapy. The patient was diagnosed in April 2016 with stage IV poorly differentiated adenocarcinoma of the right lobe of the lung and had no driver gene mutations. The primary tumor remained enlarged after 6 cycles of nedaplatin and paclitaxel, and on October 14, 2016, the patient started atezolizumab monotherapy, which showed significant reduction in both primary tumor and mediastinal lymph nodes. Subsequently, the patient developed headaches, and on May 11, 2018, right parietal lobe metastasis of the tumor was confirmed. On May 23, 2018, the patient underwent brain X-knife stereotactic radiotherapy. One month later, the patient developed cough and shortness of breath, a new nodule in the right inferior lung basement area, a mild subpleural large lesion in the left inferior lobar area, a small right-sided A small pleural effusion was noted on the right side. Continued treatment with atezolizumab resulted in a decrease in the number of nodules in the basal right lower lobe, a decrease in the number of subpleural lesions in the basal left lower lobe, and a decrease in the number of mediastinal lymph nodes. This indicates significant efficacy of atezolizumab monotherapy in the treatment of lung adenocarcinoma. IMpower130 reported an evaluation of the efficacy and safety of atezolizumab plus chemotherapy versus chemotherapy alone as primary treatment for nonsquamous non-SCLC (56). Patient inclusion criteria were age 18 years or older, histologically or cytologically confirmed diagnosis of stage IV non-squamous non-SCLC, Eastern Cooperative Oncology Group performance status of 0 or 1, and no prior stage IV chemotherapy. Patients were randomized to receive either atezolizumab plus chemotherapy (carboplatin [area under the curve 6 mg/mL/min IV every 3 weeks] plus nab-paclitaxelor chemotherapy alone. Patients were randomized in a 2:1 ratio to receive either chemotherapy alone (four or six 21-day cycles, followed by maintenance therapy). The primary endpoints were progression-free survival and overall survival in the intention-to-treat wild-type population. The results of this study showed that atezolizumab plus chemotherapy significantly improved overall and progression-free survival compared with chemotherapy alone as a primary treatment option for stage IV non-squamous non-SCLC without ALK or epidermal growth factor receptor mutations.

#### 3.1.3 Camrelizumab

Camrelizumab is a human IgG4-κ monoclonal antibody with high affinity for PD-1. Kamrelizumab binds to PD-1 with a binding affinity of up to 3 nM and has an inhibitory effect on PD-1/PD-L1 with an IC50 of 0.70 nM. Camrelizumab has antitumor activity and is well-tolerated in experimental cancers such as NSCLC (57), Hodgkin lymphoma (58) and HCC (59). The safety and efficacy of immune checkpoint inhibitors (including durvalumab, atezolizumab, nivolumab, tripalimumab, tisulizumab, cintilizumab, and camrelizumab) in patients with BMfrom SCLC were evaluated (60). The group retrospectively reviewed the medical records of patients with SCLC who received chemotherapy and radiation therapy for BMwith or without immune checkpoint inhibitors from January 2019 to January 2021 at our institution. Patients were divided into two groups: Group A received chemotherapy and radiation therapy for brain metastases; Group B received chemotherapy, radiation therapy for brain metastases, and immunotherapy for at least four cycles. Overall survival and intracranial progression-free survival were evaluated using Kaplan-Meier estimation and Cox regression modeling. The analysis showed that the intracranial objective response rate was higher in group B than in group A, but the intracranial disease control rate was similar in both groups, indicating that immunotherapy plus chemotherapy plus radiation therapy was favorably effective in patients with BM from SCLC.

#### 3.1.4 Durvalumab

Durvalumab is a humanized antibody that affects the immune response by binding to PD-L1. Durvalumab fights tumors by inhibiting the binding of PD-L1 to PD-1 and enhances the killing of tumor cells by T cells. Currently, durvalumab is FDA-approved for the treatment of lung cancer (61), esophageal cancer (62), stomach cancer (63), and prostate cancer (64). Researcher describes the therapeutic efficacy of durvalumab in a patient with stage III SCLC (65). The patient developed lung and BM after concurrent chemoradiation therapy (cCRT) and achieved complete radiologic local regression after whole brain irradiation (WBI) using the simultaneous integrated boost (SIB) technique. Durvalumab was then used as maintenance therapy. After the second dose of durvalumab, the patient developed an asymptomatic multifocal brain tumor recurrence. In contrast, with the combination of durvalumab and amlotinib, the myeloma regressed almost completely without severe toxicity. This suggests that the combination of durvalumab and amlotinib may have a synergistic effect on myeloma in previously treated SCLC patients.

#### 3.1.5 Ipilimumab

Ipilimumab is an IgG1 kappa immunoglobulin with a molecular weight of approximately 148 kDa. Ipilimumab binds to CTLA-4 and blocks the interaction of CTLA-4 with its ligand CD80/CD86. blockade of CTLA-4 increases T cell activity and proliferation, including tumor-infiltrating effector T cells, and increases growth. inhibition of CTLA-4 signaling similarly decreases regulatory T cell function and may contribute to a general increase in T cell responsiveness, including anti-tumor immune responses. Ipilimumab is used primarily for the treatment of unresectable or metastatic melanoma and for adjuvant therapy in patients with cutaneous melanoma who have undergone total lymphadenectomy, including total lymph node excision, and have localized lymph node lesions greater than 1 mm. Adjuvant therapy. Metastatic malignant melanoma has a poor prognosis and lacks effective treatment. Patients with this disease have a median survival of only 6-9 months for stage IV and a 5-year survival rate of only 10-20% (66, 67). Once the tumor has spread to the brain, only conservative treatment is available (68). Surgery and radiation therapy are effective but often cause other lesions (69). Radiation therapy, now commonly used to treat many types of brain metastases, can palliate but not eliminate lesions (4). Chemotherapy has also been applied to treat brain metastases, but with poor efficacy and short median survival (70). Chen et al (71), investigated the impact of concurrent SRS-SRT and immune checkpoint inhibitors on the prognosis and safety of patients with BM (metastatic non-SCLC, melanoma, and renal cell carcinoma). Patients receiving SRS-SRT treatment with anti-cytotoxic T lymphocyte-related protein 4 (ipilimumab) and anti-programmed cell death protein 1 receptor (nivolumab and pembrolizumab) were included. Patients using immune checkpoint inhibitors in ongoing or unreported clinical trials were excluded, and concomitant use of ICIs was defined as ICI use within 2 weeks of SRS-SRT treatment. Patients were treated with SRS-SRT, SRS-SRT without ICI, or SRS-SRT with ICI. The results of this study suggest that ICI concurrent with SRS-SRT may reduce the incidence of new BM without increasing the incidence of adverse events and may result in favorable survival outcomes. Long et al (72), evaluated the efficacy and safety of nitolizumab alone or in combination with ibritumomab in patients with active melanoma brain metastases. They randomized asymptomatic patients with BM who had never received local brain therapy to group A (nivolumab plus ibritumomab) or group B (nivolumab), while patients with BM who were refractory to local therapy and had neurological symptoms or meningeal lesions were enrolled in a non-randomized subgroup C (nivolumab). The treatment regimen consisted of nivolumab 1 mg/kg plus ibritumomab 3 mg/kg every 3 weeks for a total of 4 doses, followed by nivolumab 3 mg/kg intravenously every 2 weeks for patients in group A and nivolumab 3 mg/kg intravenously every 2 weeks for patients in group B or C. The primary endpoint was intracranial response from week 12. The primary and safety analyses were performed on an intention-to-treat basis for all patients who received at least one treatment. The results of this study showed that both the combination of nivolumab and ipilimumab and nivolumab monotherapy had a favorable effect on melanoma BM and that the combination of nivolumab and ipilimumab is applicable to asymptomatic untreated brain metastasis patients. Amaral et al (73), also reported that asymptomatic and symptomatic Amaral et al. also evaluated the efficacy of nituzumab plus ibritumomab alone or in combination with local therapy in patients with asymptomatic and symptomatic melanoma BM(MBM). Results showed no difference in OS between patients treated with BRAF and MEK inhibitors or nivolumab plus ibritumomab, and no difference in OS between initial and subsequent treatment with nituzumab plus ibritumomab in BRAF wild-type patients. In contrast, patients who received stereotactic radiosurgery or surgical local therapy had improved OS compared to patients who did not receive local therapy. Thus, the combination of nivolumab plus ibritumomab immunotherapy and stereotactic radiosurgery or surgery was found to improve OS in both asymptomatic and symptomatic MBM. Borzillo et al (74), used the CyberKnife system to compare SRT/SRS with ibritumomab (IPI) to evaluate the association and timing. They tested the correlation in 53 patients treated with RT+IPI and 10 patients treated with RT alone. Results showed that IPI combined with SRS/SRT improved LC in the treatment of MBM, but the impact and timing of both therapies on patient prognosis is unknown. Tawbi et al (75), tested the efficacy of nivolumab and ibritumomab combination therapy in patients with symptomatic MBM. The study protocol consisted of nivolumab 1 mg/kg and ipilimumab 3 mg/kg IV every 3 weeks for 4 doses, followed by nivolumab 3 mg/kg IV every 2 weeks for up to 2 years until disease progression or unacceptable toxicity. The primary endpoint was to assess the intracranial clinical response rate in all patients treated. Secondary endpoints were intracranial progression-free survival and overall survival. The results of this study showed that the combination of nituzumab and ibritumomab improved progression-free survival in patients with symptomatic MBM without causing serious side effects. A multivariate predictive model of response and survival to anti-programmed cell death protein-1 (anti-PD-1) monotherapy or in combination with anti-cytotoxic T-cell lymphocyte-4 (ipilimumab [IPI]; anti-PD-1 + IPI) was developed in metastatic melanoma (76). Study endpoints were objective response rate (ORR), progression-free survival (PFS), and overall survival (OS). And the area under the curve of the final model predicting ORR for immunotherapy-treated patients was 0.71, indicating that the model can predict response and survival outcomes for metastatic melanoma patients receiving immunotherapy.

#### 3.1.6 Nivolumab

**Figure 4:**
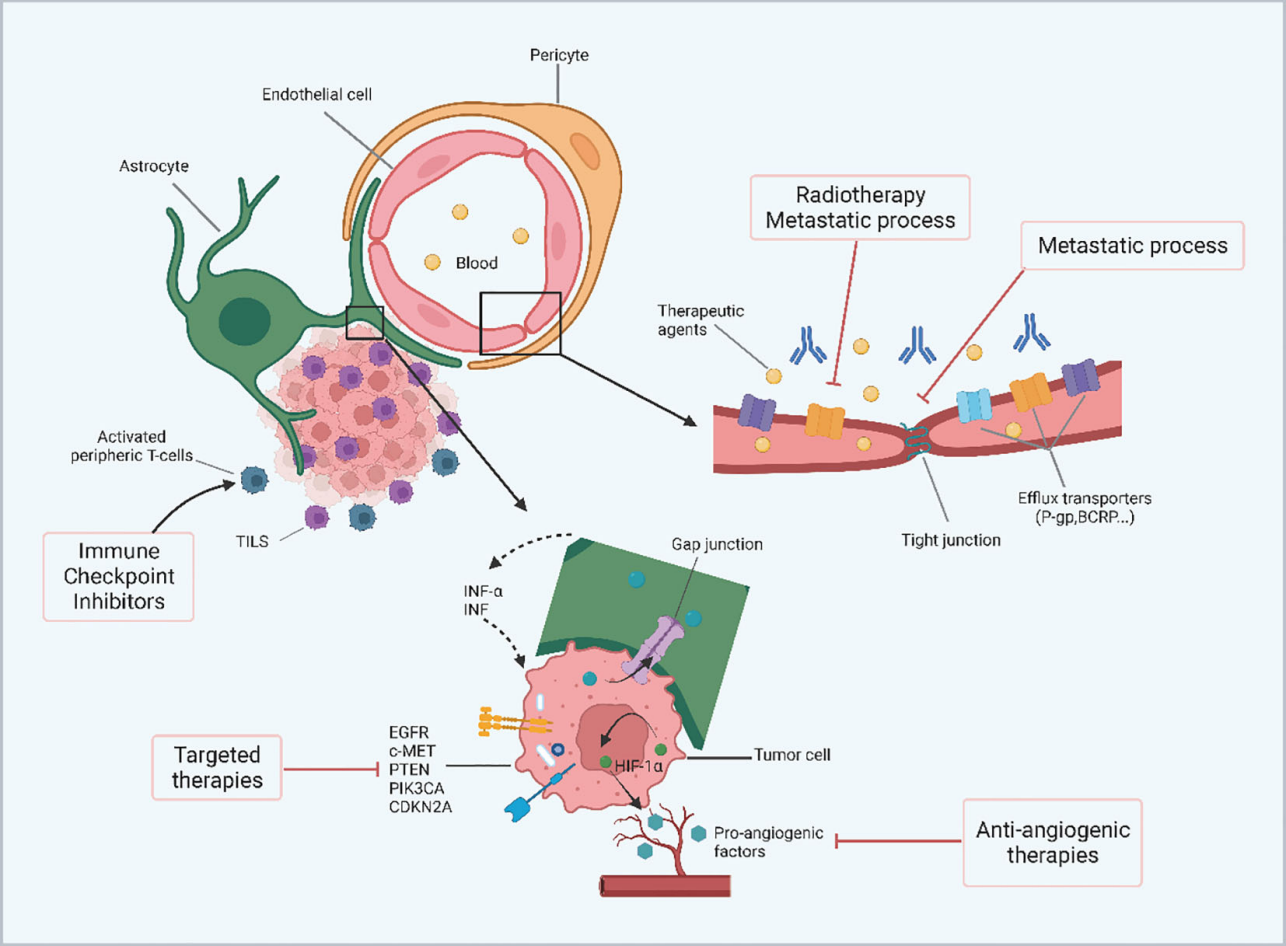
The overview of the challenges of brain metastasis from renal cell carcinoma. In the microenvironment of renal cancer brain metastases, the BBB can limit the permeability of therapeutic agents. The number of efflux transporters is significantly reduced during metastasis and radiotherapy thereby increasing the permeability of peripheral molecules. In addition, tight junctions are impaired by metastatic development leading to increased permeability of the blood-brain barrier. Gap junctions may allow metabolite transfer between renal cancer cells and astrocytes and induce secretion of INF-α and TNF by astrocytes, leading to chemoresistance. The molecular characteristics of renal cancer cells, including highly angiogenic features, molecular inconsistencies between primary and brain metastases, and inherent radio resistance also affect the outcome of renal cancer brain metastases. (Created with BioRender.com).

Nivolumab is an immunotherapeutic drug that targets PD-1 and inhibits the interaction between PD-1 and its ligand PD-L1, thereby restoring immune cell activity and strengthening the body’s defense against cancer. Nivolumab is widely used to treat a variety of cancers and has shown good efficac (77). Unresectable or metastatic melanoma, melanoma as adjuvant therapy, resectable or metastatic non-SCLC, SCLC, advanced renal cell carcinoma, classical Hodgkin lymphoma, head and neck squamous cell carcinoma, urothelial carcinoma, metastatic colorectal cancer with high microsatellite instability or defective mismatch repair, liver cell carcinoma, esophageal cancer, and others with high microsatellite instability or defective mismatch repair. Chen et al (71), investigated the impact of concurrent administration of SRS-SRT and immune checkpoint inhibitors on prognosis and safety in patients with BM (metastatic non-SCLC, melanoma, and renal cell carcinoma). Patients receiving SRS-SRT treatment with anti-cytotoxic T lymphocyte-related protein 4 (ipilimumab) and anti-programmed cell death protein 1 receptor (nivolumab and pembrolizumab) were included. Patients using immune checkpoint inhibitors in ongoing or unreported clinical trials were excluded, and concomitant use of ICIs was defined as ICI use within 2 weeks of SRS-SRT treatment. Patients were treated with SRS-SRT, SRS-SRT without ICI, or SRS-SRT with ICI. The results of this study showed that ICI concurrent with SRS-SRT may reduce the incidence of new BM without increasing the incidence of adverse events and may result in favorable survival outcomes. Crinò et al (78), reported the efficacy and safety of nivolumab in nonsquamous NSCLC. In their study, nivolumab was indicated for patients with stage IIIB/IV non-squamous NSCLC whose disease had progressed after at least one prior therapy. Patients with brain metastases, on the other hand, were included as long as they were asymptomatic, neurologically stable, had discontinued corticosteroids, or their prednisone dose was stable or reduced to less than 10 mg per day. The results of this study suggest that patients with BMfrom non-squamous NSCLC are asymptomatic or have controlled brain metastases. Renal cell carcinoma accounts for approximately 4% of all solid tumors, with an incidence of approximately 16.1 per 100,000 population; approximately 1/3 of RCC patients are in an advanced stage at diagnosis (79, 80). The first-line treatment for advanced renal cell carcinoma is targeted therapy combined with immunotherapy, and metastasectomy, radiofrequency ablation, and targeted therapy are the preferred treatment for patients with oligometastatic or low tumor burden trans metastatic renal cell carcinoma (mRCC) (81). Although these therapies help prolong OS in patients with mRCC, the side effects of drug therapy affect patients’ quality of life. An overview of the challenges of brain metastasis in renal cell carcinoma is shown in **Figure 4**. Flippot et al (82), evaluated the activity of nivolumab in patients with brain metastatic clear cell renal cell carcinoma (ccRCC) after failure of angiogenic therapy. The study population consisted of patients with previously treated or untreated brain metastases. The primary endpoint was intracranial response efficiency in patients. Study results showed limited activity of nivolumab in patients with untreated ccRCC brain metastases. Brain imaging and treatment of the lesion should be considered before using immune checkpoint inhibitors in patients with metastatic ccRCC. Reardon et al (83) examined the role and value of bevacizumab and nivolumab monotherapy in improving survival in patients with recurrent glioblastoma. The study recruited 439 patients (369 of whom were randomized) with first recurrence of glioblastoma after treatment with standard radiation therapy and temozolomide. Patients were randomized 1:1 to either nivolumab 3 mg/kg or bevacizumab 10 mg/kg every 2 weeks until disease progression, unacceptable toxicity, or death was confirmed. The primary endpoint was OS. The results of this study suggest that overall mOS values for nivolumab and bevacizumab in patients with recurrent glioblastoma are similar, and the safety profile of nivolumab in patients with glioblastoma is consistent with that of other tumor types. The CheckMate 920 trial investigated the safety and efficacy of nivolumab plus ipilimumab in patients with advanced renal cell carcinoma (aRCC) and brain metastases. The study revealed a 32% objective response rate (ORR) in response-evaluable patients, with a median duration of response (DOR) of 24.0 months and a median time to response (TTR) of 2.8 months. Some patients experienced intracranial progression, and the safety profile of the treatment regimen, including immune-mediated adverse events, was assessed (83). 

#### 3.1.7 Pembrolizumab

**Figure 5:**
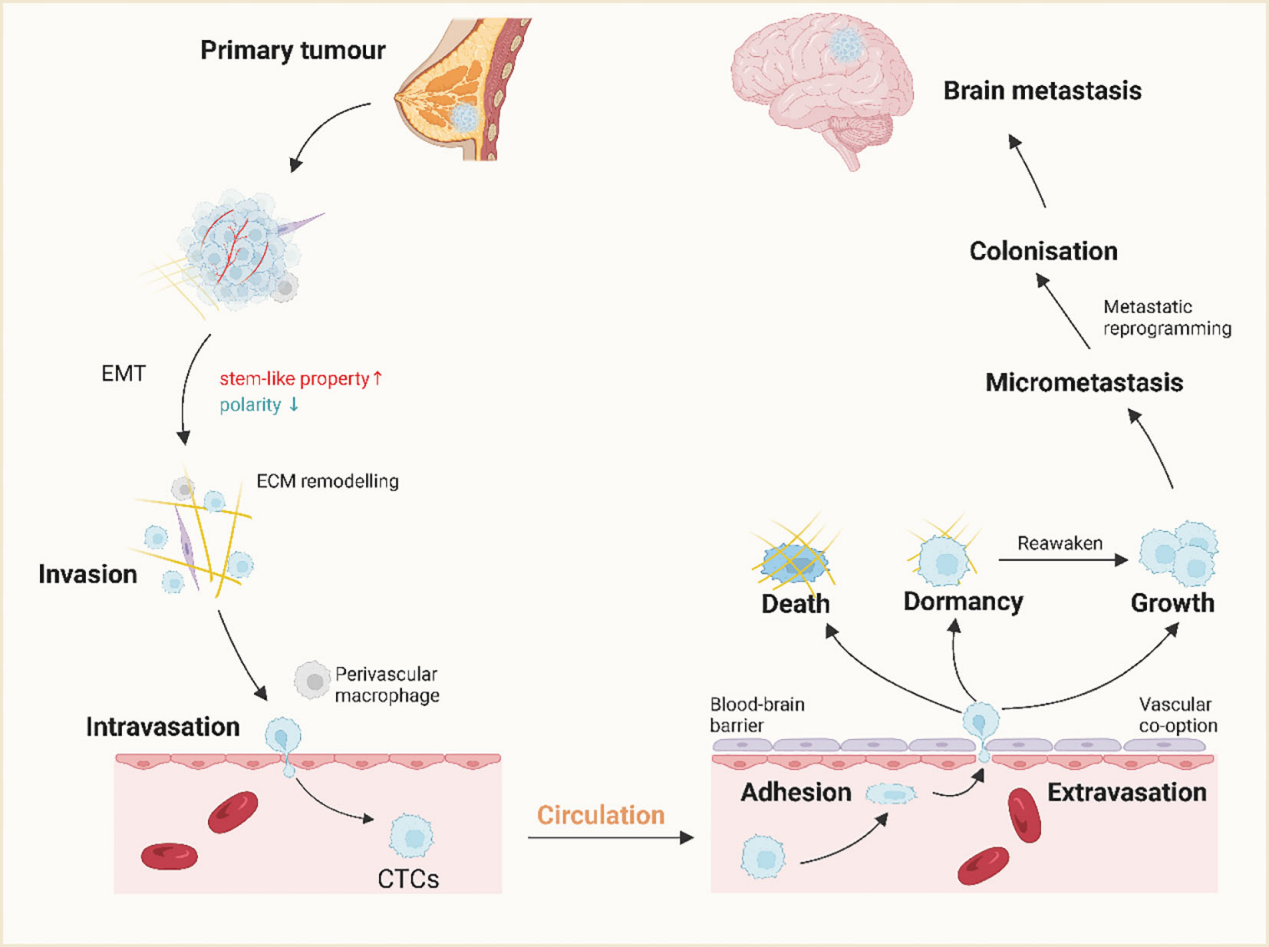
The mechanism of brain metastasis of breast cancer. A small population of breast cancer cells at the primary site acquires stem cell-like properties and, through epithelial-mesenchymal transition (EMT), invasive properties. Invasive breast cancer cells infiltrate the surrounding tissue through ECM remodeling and become circulating tumor cells (CTCs) with the help of perivascular macrophages and interactions with vascular endothelial cells (ECs).CTCs spread throughout the body through the bloodstream and cross the BBB through extravasation after adhering to endothelial cells in the brain. The majority of the cells die or become dormant, while a small number of cells proliferate in this new microenvironment. this new microenvironment proliferate. In addition, dormant cells are often reawakened under certain conditions and participate in colonization, leading to tumor recurrence. (Created with BioRender.com).

Pembrolizumab exerts its antitumor effects by inhibiting the binding of PD-1 receptors on immune cells to PD-L1 on tumor cells, thereby restoring the ability of immune cells to attack tumors. pembrolizumab has been used in the treatment of many different Nasopharyngeal carcinoma (84) with broad indications for the treatment of many different types of malignancies. Studies have shown that pembrolizumab is an alternative to cytotoxic chemotherapy as first-line therapy in patients with PD-L1 tumor percentage scores of 50% or greater. The study reported the use of pembrolizumab in patients with giant cell lung cancer (85). A 69-year-old female patient with giant cell lung cancer, clinically classified as IVB (T2bN0M1c, BRA) and with a high percentage of tumors expressing PD-L1, received stereotactic radiotherapy targeting two cerebellar metastases, followed by immunotherapy with an anti-PD-1 antibody (pembrolizumab) for four treatment cycles. The tumor shrank significantly after 4 cycles of treatment. However, treatment was discontinued due to renal dysfunction. This suggests that pembrolizumab combined with radiotherapy also has a favorable therapeutic effect in giant cell lung cancer. reported the management of a patient with multiple metastases of NSCLC with exon 19 deletion and PD-L1 deletion. Pembrolizumab plus chemotherapy and SBRT were then initiated for the supraclavicular metastases and spinal cord lesions; examination after four cycles showed resolution of adenopathy, reduction in lung mass, liver and spinal cord lesions, and no lesions or new metastases were detected on brain MRI. The patient then continued treatment with pembrolizumab plus pemetrexed for almost a year and is now in good disease control. A study was conducting a phase II trial of pembrolizumab in untreated NSCLC or melanoma patients with BM to investigate the activity of PD-1 blocking agents in the central nervous system (86). Cohort 1 consisted of patients with PD-L1 ≥1% and cohort 2 consisted of patients with PD-L1 <1% or not evaluable. The primary endpoint was the proportion of patients who achieved a response in brain metastases. The results of this study showed that pembrolizumab is effective for NSCLC BM with PD-L1 expression of 1% or greater and is safe in some untreated patients with brain metastases. Breast cancer is the most common malignancy in women worldwide, and the incidence of BM from breast cancer is on the rise, ranging from 5% to 21% (87, 88), as new therapeutic agents and imaging techniques advance (**Figure 5**). The incidence of BM from breast cancer is highest in HER2-positive types, accounting for about 30% to 55% (89, 90). There are mainly parenchymal and meningeal metastases, with parenchymal metastases being more common (91, 92). The prognosis for BM from breast cancer is usually very poor because the BBB severely limits the entry of most chemotherapeutic agents into the nervous system (93, 94). Local radiation therapy and surgical treatment can slow the progression of the disease, but it is difficult to completely kill the cancer cells in the body (95, 96). Therefore, the development of systemic therapeutics and the selection of new therapies are crucial for the treatment of brain metastasis of breast cancer. Wu et al (97),. reported on the combination of anti-estrogenic drugs and immunotherapy in patients with HR-positive metastatic breast cancer. The first patient was a patient with recurrent breast cancer with ovarian and BM after endocrine therapy. After surgery for the ovarian lesions and three cycles of chemotherapy, a high degree of T-cell receptor (TCR) complexes were observed in the tumor. The patient then received a combination of trazodone and pembrolizumab. The patient achieved a partial response and had a PFS of more than 21 months; the second patient was a breast cancer patient with multiple bone metastases. The second patient had multiple bone metastases and was treated with a combination of tamoxifen and pembrolizumab because the combination of radiation and chemotherapy was ineffective. Another patient with BM from lung cancer underwent local resection of two BM by SRS and received systemic immunochemotherapy consisting of four cycles of cisplatin, pemetrexed, and pembrolizumab. The patient then underwent left posterolateral thoracotomy, left lower lobe expansion resection, segment 1 wedge resection, and systematic clearance of hilar, mediastinal, and interlobar lymph nodes. Maintenance therapy with pembrolizumab was resumed postoperatively and was uneventful for 2 years. Thirty-five months after the initial diagnosis, CT scan of the chest and abdomen and MRI of the cranium showed no signs of local recurrence or metastasis. The ORR (objective response rate) of brain metastasis when using pembrolizumab was found to be 28.6%. The PFS (progression-free survival) was reported to be 4.0 months. These outcomes suggest that pembrolizumab may have some efficacy in the treatment of brain metastasis. However, further research and clinical trials are needed to establish its effectiveness and safety in this specific patient population (98).

#### 3.1.8 Sintilimab

Sintilimab is a novel immunotherapeutic agent that is a humanized monoclonal antibody. Sintilimab is widely used to treat a variety of malignancies, including but not limited to non-SCLC, melanoma, ESCC, renal cell carcinoma, and bladder cancer (99, 100). It is considered an innovative therapy that has the potential to change the landscape of conventional tumor treatment. Nong et al. Nong et al (101), reported the diagnosis and treatment with sintilimab in a patient with lung adenocarcinoma with brain metastases. The patient had an in-frame insertion of epidermal growth factor receptor exon 20 and was treated with pemetrexed and carboplatin plus the programmed cell death-1 inhibitor sintilimab After six cycles of treatment, the patient received sintilimab plus pemetrexed for patients received maintenance therapy every 3 weeks with sintilimab plus pemetrexed, which was effective without toxicity. This suggests an important role for sintilimab in patients with brain metastatic NSCLC who have an insertional mutation in exon 20 of the epidermal growth factor receptor.

#### 3.1.9 Tisulizumab

Tislelizumab is a new generation targeted immunotherapy drug widely used to treat many malignancies. It is a humanized monoclonal antibody that strengthens a patient’s own immune system to fight tumor cells by targeting the immune checkpoint PD-1. It has shown excellent antitumor activity in a wide range of cancer types (102), including non-SCLC, melanoma, renal cell carcinoma, esophageal cancer, and nasopharyngeal cancer. The use of tislelizumab can further improve survival and quality of life for patients. Fu et al (103),. reported the progress of tislelizumab in a patient with invasive lung adenocarcinoma with brain metastases. The patient underwent right upper lung lobectomy and lymph node dissection. Postoperative pathology revealed invasive adenocarcinoma (alveolar, papillary, and enhancing) with pleural invasion, staged cT0N0M1c, stage IVC. POLE and TP53 mutations were found. The patient then received two cycles of combination therapy with pemetrexed + carboplatin + bevacizumab + tislelizumab. After two cycles of treatment, the patient’s intracranial metastases became smaller; after four cycles of combination therapy, the patient’s metastases completely resolved After four cycles of combination therapy, the patient’s metastases had completely disappeared. The patient then received two cycles of consolidation therapy with tislelizumab, pemetrexed, and bevacizumab. 6 cycles of treatment later, the patient felt fatigue and anorexia. Treatment was then switched to tislelizumab and bevacizumab for six cycles to date. The patient responded well and had no treatment-related adverse events 11 months after starting the combination.

#### 3.1.10 Toripalimab

Toripalimab is a PD-1 antibody that inhibits the immune escape mechanism of melanoma cells by targeting the immune checkpoint PD-1, a membrane surface receptor that regulates immune response homeostasis and prevents over-activated immune cells from attacking normal tissue. In melanoma, tumor cells normally overexpress PD-L1, which binds to PD-L1 to inhibit immune cell function and evade immune attack. Reported on the diagnosis and treatment with toripalimab in a patient with SCLC with brain metastases (104). The patient was diagnosed with localized stage small cell carcinoma of the left lower lung and received 6 cycles of initial chemotherapy with etoposide and nedaplatin followed by adaptive intensity modulated radiation therapy (IMRT) to the thorax and prophylactic brain radiation therapy to achieve CR. Approximately 3 months after completion of radiotherapy, chest CT and brain-enhanced MRI confirmed that CR was sustained. Approximately 6 months after completion of radiotherapy, a cranial-weighted MRI showed metastasis in the left cerebellar hemisphere. The patient was then treated with IMRT and anlotinib; at the end of IMRT, irinotecan and lopressor were added to anlotinib. Due to grade 3 adverse events, patients received 3 cycles of maintenance therapy with sindilizumab plus anlotinib (104). However, 2.5 months after achieving CR, the BM recurred. Because the recurrent lesion was small and asymptomatic, treatment with sindilizumab in combination with erlotinib was continued for 3 more cycles. A skull-weighted MRI showed no change in the target lesion. The physician then switched from sindilizumab to toripalimab. After two cycles of treatment with toripalimab in combination with anlotinib, the recurrent BM reached CR and were maintained for 6 months. In conclusion, the safety profile of toripalimab in combination with erlotinib was favorable and no serious adverse events were observed during treatment.

### 3.2 Immune cell therapy

Lymphocytes that possess cytotoxic abilities *in vivo* encompass natural killer cells and cytotoxic T cells, both of which can effectively counteract tumor cell proliferation. Empirical evidence suggests that several hundred lymphocytes are required to combat a single tumor cell. Hence, a larger population of lymphocytes confers a greater capacity for tumor cell elimination and inhibition of tumor cell production. This fundamental principle forms the basis of cellular immunotherapy. Presently, cellular biopharmacotherapy, exemplified by cellular immunotherapy, represents a significant advancement in the field of tumor biotherapy. Adoptive cellular immunotherapy (ACI) is the accepted nomenclature for this modality and entails the infusion of immune cells with antitumor properties (both specific and nonspecific) into tumor patients, either for direct tumor eradication or to induce the patient’s immune response to target tumor cells (105). Clinically, ACI entails the administration of autologous or allogeneic immune effector cells that have been activated *in vitro*, thereby inducing tumor cell death within the patient’s body (106, 107). In recent years, cellular immunotherapy has emerged as a vibrant domain within tumor biotherapy, specifically suited for patients with compromised cellular immunity, particularly those afflicted with hematologic and immune system malignancies, such as those arising after intense chemotherapy, radiotherapy, bone marrow transplantation, or viral infections leading to depletion and dysfunction of immune cells (108, 109). Cellular immunotherapy possesses the ability to selectively suppress and eliminate tumor cells, independent of the patient’s inherent immune function, and can be effectively combined with radiation therapy and chemotherapy. The efficacy, specificity, overall therapeutic effectiveness, and side effect profile of this approach have shown progressive enhancements during the evolutionary stages of LAK, TIL, CD3AK, CIK, DC-CIK, and EAAL (110, 111).

### 3.3 Tumor vaccine

Tumor vaccines have gained significant attention in recent years as a focal point of research in the medical field. The fundamental concept behind tumor vaccines involves the administration of tumor antigens into the patient’s system by various means, such as tumor cells, tumor-related proteins and peptides, and genetic material encoding tumor antigens. This approach is aimed at counteracting the immune-suppressive environment induced by the tumor, enhancing immunogenicity, and stimulating the patient’s endogenous immune response. Activation and stimulation of both cellular and humoral immune responses in the body are crucial components in achieving the ultimate goal of controlling or eradicating tumors (112, 113). There are three primary classifications of tumor vaccines: prophylactic tumor vaccines, therapeutic tumor vaccines, and immuno-cellular therapy vaccines (114). Prophylactic tumor vaccines are primarily utilized to prevent the development of specific malignancies, such as cervical and liver cancer vaccines. In contrast, therapeutic tumor vaccines are designed for the treatment of patients with existing tumors. Examples of therapeutic tumor vaccines encompass tumor cell vaccines and tumor-associated antigen vaccines. Finally, immuno-cellular therapeutic vaccines involve the manipulation of the patient’s immune cells to combat tumors, including tumor-infiltrating lymphocyte vaccines and dendritic cell vaccines.

As scientific knowledge and technological capabilities continue to progress, the categorization of tumor vaccines is evolving, presenting new possibilities and optimism for tumor prevention and treatment. Current research predominantly concentrates on the advancement and assessment of tumor vaccines as a potential therapeutic modality for a range of malignancies. In recent times, there have been significant advancements in comprehending the mechanisms through which tumors evade the immune system and devising strategies to overcome these challenges. Various types of tumor vaccines, such as peptide-based, dendritic cell-based, and whole tumor cell-based vaccines, are undergoing scrutiny in preclinical and clinical trials. These vaccines are designed to provoke a targeted immune response against tumor antigens, with the objective of facilitating the specific destruction of tumor cells, averting tumor recurrence, and enhancing patient outcomes.

While early-phase trials have shown promise, further exploration and refinement of vaccine design, delivery modalities, and patient selection are imperative to amplify their effectiveness and delineate their role in cancer management. Consequently, ongoing research in this domain holds substantial promise for introducing novel therapeutic avenues to combat malignancies and elevate the quality of patient care.

In summary, immune checkpoint inhibitors, such as CTLA-4 inhibitors (e.g., Ipilimumab), PD-1 inhibitors (e.g., Pembrolizumab, Nivolumab), PD-L1 inhibitors (e.g., Atezolizumab, Durvalumab), LAG-3 inhibitors (e.g., Relatlimab), and TIM-3 inhibitors (e.g., Sabatolimab), have distinct characteristics and mechanisms of action. These inhibitors target specific receptors on T cells or ligands on cancer cells, aiming to enhance the immune response against cancer by restoring T cell activity, promoting anti-tumor immune responses, and potentially reversing T cell exhaustion. Immune checkpoint inhibitors play a vital role in cancer immunotherapy, offering potential in improving the body’s ability to combat cancer (**Table 2**). Immune checkpoint inhibitors are pivotal in modulating the immune response directed towards cancer cells through the targeting of specific regulatory checkpoints governing T cell functionality. Integral to cancer immunotherapy, these agents exhibit significant potential in bolstering the host’s anti-cancer defenses.

**Table 2:** Immune checkpoint inhibitors along with the biological characteristics.

Classification	Representatives	Mechanisms
CTLA-4 Inhibitors	Ipilimumab	Blocks CTLA-4 receptor on T cells, enhancing immune response against cancer cells.
PD-1 Inhibitors	Pembrolizumab, Nivolumab	Targets PD-1 receptor on T cells, prevents interaction with PD-L1 on cancer cells, restores T cell activity against tumors.
PD-L1 Inhibitors	Atezolizumab, Durvalumab	Targets PD-L1 ligand on cancer cells, disrupts binding with PD-1 on T cells, promotes anti-tumor immune responses.
LAG-3 Inhibitors	Relatlimab	Targets LAG-3 receptor on T cells, enhances T cell function and anti-tumor immunity.
TIM-3 Inhibitors	Sabatolimab	Blocks TIM-3 receptor on T cells, potentially reverses T cell exhaustion, improves anti-tumor responses.

## 4 Discussion

The tumor microenvironment (TME) is a complex and dynamic system that plays a crucial role in tumor development and growth. It consists of four major components: non-tumor cells, extracellular matrix, vasculature, and soluble products (115, 116). Non-tumor cells in the TME include immune cells, fibroblasts, endothelial cells, and neurons. The extracellular matrix provides structural support for cells and regulates their functions through a network of proteins. The vasculature supplies oxygen and nutrients to tumor cells, often forming a dense network around tumors. Soluble products, such as chemokines, within the TME significantly influence cellular activities (117). The interactions among these components have profound effects on tumorigenesis, metastasis, and drug resistance, ultimately impacting the metabolic patterns and immune responses within the TME. Brain metastases (BM) are a common and challenging complication in cancer patients, affecting over 10% of patients at diagnosis and escalating to 30-40% during disease progression (118, 119). Patients with BM have a grim prognosis, marked by high mortality rates, poor quality of life, and a median overall survival of merely 4-6 months. The blood-brain barrier (BBB) poses challenges for the treatment of BM by limiting the efficacy of systemic chemotherapy. Current treatment options for BM include surgical resection, stereotactic radiosurgery, and whole-brain radiation therapy. However, their effectiveness varies depending on the number and size of metastatic brain lesions, with whole-brain radiation therapy typically yielding response rates between 50% and 75%, and survival rates ranging from 4 to 9 months Recent advances in tumor immunology research have led to the development of targeted therapies, such as CTLA-4 and PD-1/PD-L1 inhibitors, which have shown promising efficacy rates compared to traditional chemotherapeutic agents (120, 121). However, the effectiveness of immunotherapy alone may be limited due to immunosuppressive factors in tumor patients. Therefore, combination therapies involving tumor immunotherapy and other treatment modalities have become a future direction in cancer treatment (122, 123). Although immunotherapy has demonstrated significant benefits in treating advanced tumors, its efficacy is limited to certain tumor types, and individual differences may result in poor response rates. Immune-related complications are also common. Therefore, efforts to improve the efficiency and reduce the risk of tumor recurrence of immunotherapy, particularly PD-1/PD-L1 inhibitors, are needed (124, 125). Predictive markers and an understanding of drug resistance mechanisms are crucial for treatment selection and prognostic assessment.

The presence of the BBB poses a challenge for conventional chemotherapeutic agents to effectively reach brain metastases. The successful application of immune checkpoint inhibitors (ICIs) in patients with brain metastatic cancer has raised expectations for the potential of immunotherapy in the treatment of primary and metastatic brain cancer. However, the clinical implementation of immunotherapy in patients with brain metastases faces substantial obstacles due to the lack of robust predictors and appropriate animal models for evaluating efficacy. A comprehensive understanding of the biological underpinnings and specific mechanisms of immunosuppression in brain metastatic cancer is imperative for the development of novel immunological interventions. Clinical trials are needed to establish the effectiveness of immunotherapy in treating brain metastatic cancer and to identify precise biomarkers for patient selection. Furthermore, the heterogeneity of brain metastases and the limited infiltration of immune cells present challenges for effective immunotherapy. Strategies to enhance the penetration of immunotherapeutic agents through the BBB and to increase immune cell recruitment and infiltration into brain metastases are being explored. Resistance mechanisms, such as immune checkpoint upregulation and alterations in antigen presentation, can also develop in brain metastases. Combination therapies that target multiple resistance mechanisms and immunotherapies are necessary to overcome resistance and improve treatment outcomes. Managing immune-related adverse events and developing more targeted and selective immunotherapies are essential for the safe and effective use of immunotherapy in brain metastases. Combination therapies, targeted delivery systems, and personalized immunotherapies are being investigated to improve the efficacy and safety of immunotherapy for brain metastases.

In conclusion, immunotherapy holds promise as a treatment approach for brain metastases. However, challenges related to the BBB, tumor heterogeneity, limited immune cell infiltration, resistance mechanisms, and immune-related adverse events need to be addressed to optimize the efficacy and safety of immunotherapy in patients with brain metastases. Various strategies, such as enhanced BBB penetration, combination therapies, and personalized immunotherapies, are being explored to overcome these challenges and improve treatment outcomes.

## Author contributions

TL: Writing – original draft. SS: Conceptualization, Investigation, Writing – original draft. YL: Writing – original draft. YZ: Writing – review & editing. LW: Writing – original draft, Writing – review & editing.
